# Natural-Derived Polysaccharides From Plants, Mushrooms, and Seaweeds for the Treatment of Inflammatory Bowel Disease

**DOI:** 10.3389/fphar.2021.651813

**Published:** 2021-04-26

**Authors:** Cailan Li, Guosong Wu, Hualang Zhao, Na Dong, Bowen Wu, Yujia Chen, Qiang Lu

**Affiliations:** ^1^ Department of Pharmacology, Zunyi Medical University, Zhuhai Campus, Zhuhai, China; ^2^ Pharmacy Department, Baiyun Branch of Nanfang Hospital of Southern Medical University, Guangzhou, China; ^3^ Department of Pharmaceutical Sciences, Zunyi Medical University, Zhuhai Campus, Zhuhai, China

**Keywords:** inflammatory bowel disease, polysaccharides, therapeutic effects, action mechanism, plants, mushrooms, seaweeds

## Abstract

Inflammatory bowel disease (IBD) is a chronic inflammatory disease impairing the gastrointestinal tract, and its incidence and prevalence have been increasing over time worldwide. IBD greatly reduces peoples' quality of life and results in several life-threatening complications, including polyp, toxic colonic dilatation, intestinal perforation, gastrointestinal bleeding, and cancerization. The current therapies for IBD mainly include drugs for noncritical patients and operation for critical patients. However, continuous use of these drugs causes serious side effects and increased drug resistance, and the demand of effective and affordable drugs with minimal side effects for IBD sufferers is urgent. Natural-derived polysaccharides are becoming a research hotspot for their therapeutic effects on IBD. This study focuses on the research progress of various natural polysaccharides from plants, seaweeds, and mushrooms for the treatment of IBD during recent 20 years. Regulation of oxidative stress, inflammatory status, gut microbiota, and immune system and protection of the intestinal epithelial barrier function are the underlying mechanisms for the natural-derived polysaccharides to treat IBD. The excellent efficacy and safety of polysaccharides make them promising candidates for IBD therapy.

## Introduction

Inflammatory bowel disease (IBD) includes two chronic idiopathic inflammatory diseases: Crohn’s disease (CD) and ulcerative colitis (UC), which have become prevalent all over the world (Ananthakrishnan et al., 2019). According to statistics, the prevalence of IBD is the highest in North America, Europe, and Oceania, which exceeded 0.3% of the population (Ng et al., 2017). In contrast, the incidence and prevalence of IBD in Asia and Africa was relatively rare (Kaplan and Ng, 2016; Kaplan and Ng, 2017). However, with westernized diet and lifestyle, a wave of rapidly rising incidence has followed (Mak et al., 2020). The annual direct and indirect expenses associated with IBD are assessed to be as high as €4.6–5.6 billion in Europe and US$6 billion in the United States of America (Kaplan, 2015).

**FIGURE 1:**
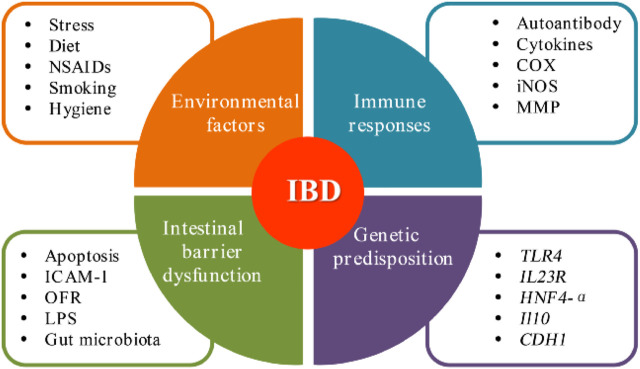
Current overview on the pathogenesis of IBD.

IBD is a chronic inflammatory disease of the gastrointestinal tract, which is characterized by long course, difficulty in curing, low quality of life, and high risk of canceration (Sairenji et al., 2017). At present, IBD has been listed as one of the modern refractory diseases by the World Health Organization. The main clinical symptom of IBD is bloody diarrhea, abdominal pain, hematochezia and emesis; some patients have extraintestinal manifestations, such as arthritis, iridocyclitis, hepatosis, and eye and skin lesion (Vavricka et al., 2015; Ribaldone et al., 2019). No sex predominance exists in IBD, and the peak age of disease onset is between ages 17 and 40 years (Kotze et al., 2020). Although the cause of IBD remains unknown, studies have provided evidence that the pathogenesis (Figure 1) of IBD is multifactorial, mainly including genetic predisposition, epithelial barrier defects, dysregulated immune responses, and environmental factors (Guan, 2019; Li C. L. et al., 2020). Modern medical therapy for patients with IBD mainly involves aminosalicylic acids, glucocorticoids, immunosuppressants, and biological agents, which can control its symptoms (Eberhardson et al., 2019). However, many disadvantages (Table 1) are increasingly prominent, such as severe side effects, easy recurrence, increased drug resistance, and high price (Moreau and Mas, 2015). Therefore, scientists are constantly devoting themselves to the development of more safe and effective drugs for treating IBD.

**TABLE 1:** Current drugs and their disadvantages for the treatment of IBD.

Type	Drug	Limitation	Reference
5-amino salicylic acids	Balsalazide	There are certain adverse reactions such as diarrhea, stomachache, nausea, and emesis. The new-type 5-amino salicylic acids including mesalazine and olsalazine are expensive	Caprilli et al. (2009); Rosenberg and Peppercorn (2010)
	Sulfasalazine		
	Mesalazine		
	Olsalazine		
Glucocorticoids	Hydrocortisone	Short-term treatment has a good effect, while long-term application may lead to adverse reactions such as moon-shaped face, weakened immunity, and acne, whose efficacy and safety are difficult to be guaranteed	Ren et al. (2015); Rosenberg and Peppercorn (2010)
	Prednisone		
	BDP		
	Budesonide		
Immunosuppressants	Azathioprine	The effect takes a long time. The mechanism of action will lead to the inhibition of the body's normal immune response. Long-term application may cause liver and kidney damage	Falasco et al. (2002); Mao et al. (2017)
	6-mercapopurine		
	Cyclosporine A		
Biologicals	Infliximab	These drugs also have some adverse reactions and are expensive	Danese et al. (2014); Freeman (2012)
	Adalimumab		

**FIGURE 2:**
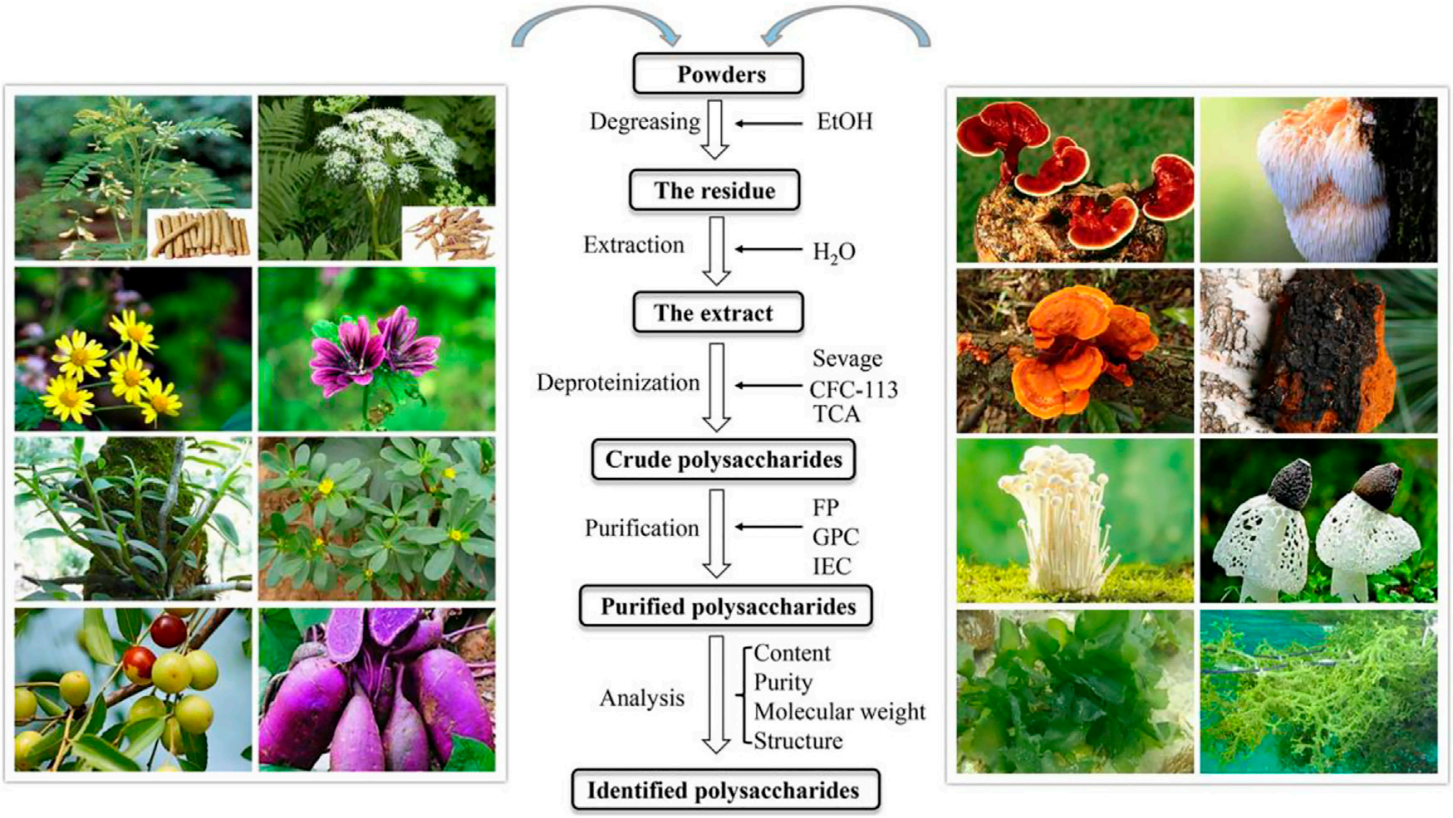
Glance at the natural sources of polysaccharides for the treatment of IBD in the past decades and the essential steps on the isolation and purification of them.

Polysaccharides are polymeric carbohydrates consisting of at least 10 monosaccharides with glycoside bonds (Ferreira et al., 2015). They range in structure from linear to highly branch. Generally, there are more than 100 monosaccharides in polysaccharides, and even as many as thousands, with great different properties (Yu et al., 2018). Polysaccharides are not only the supporting tissues and storage nutrients of animals and plants but also possess rich physiological activities, such as immune regulation (Tabarsa et al., 2020), antitumor (Khan et al., 2019), anti-oxidation (Ma et al., 2020), and so on. Notably, a large number of studies have shown that polysaccharides from different sources exerted significant inhibitory effects on IBD (Figure 2). In this study, the research progress of natural polysaccharides for the treatment of IBD was reviewed. It is hoped that it will provide inspiration for the researchers to design, research, and develop new drugs for IBD therapy.

## Methods

To identify the studies related to the effect and mechanism of natural-derived polysaccharides against IBD, we referred to the published articles in the following academic databases from the creation date to August 2020: Google Scholar, Web of Science (WOS), PubMed, and Embase. In the literature retrieval, the following search terms were adopted in combination (“polysaccharide” OR “polysaccharose”) AND (“inflammatory bowel disease” OR “IBD,” “ulcerative colitis” OR “colitis” OR “ulcer colitis” OR “UC,” OR “Crohn’s disease” OR “CD”). All articles with abstracts were considered.

After searching, the acquired articles were carefully screened. First, the articles on the effect and mechanism of natural-derived polysaccharides against IBD were initially selected through reading the titles and abstracts. Second, for the articles that cannot be identified through preliminary screening, the full text was further examined. Finally, all articles that fit the topic were imported into EndNote as the supporting resources of this review.

## Polysaccharides From Plants

Due to the wide variety, large amount, and easy access, terrestrial plants have always been the most important natural resources for human survival. The original sources of food and medicine for humans also mainly come from terrestrial plants. At present, the researches on bioactive components mainly focus on edible and medicinal plants (Atanasov et al., 2015). Significantly, there are many studies on plant polysaccharides against IBD. Tables 2, 3 listed the chemical and pharmacological information of polysaccharides from plants for the treatment of IBD, respectively.

**TABLE 2:** Monosaccharide composition, molecular weight, and main glycosidic bond of polysaccharides from natural sources.

Name	Source	Monosaccharide composition	M.W. (Da)	Main glycosidic bond	Reference
**Polysaccharides from edible and medicinal plants**					
APS	Roots of *Astragalus membranaceus*	Rha, Glc, Gal, and Ara in a ratio of 1.00:18.45:3.53:7.11	NA	NA	Lv et al. (2017)
SP1-1	Roots of *Scutellaria baicalensis*	Man, Rib, GluA, Glc, Xyl, and Ara in a ratio of 2.14:3.61:1.00:2.86:5.98:36.39	4.56 × 10^5^	NA	Cui et al. (2019)
ASP	Roots of *Angelica sinensis*	GluA, Glc, Ara, and Gal in a ratio of 1.00:1.70:1.85:5.02	8.00 × 10^4^	(1→3)-linked Gal*p*, (1→6)-linked Gal*p,* and 2-OMe-(1→6)-linked Gal*p*	Cheng et al. (2020)
DOPS	Stems of *Dendrobium officinale*	Man, Glc, and Ara in a ratio of 5.55:1.00:0.12	3.94 × 10^5^	(1→4)-linked *β*-D-mannopyranosyl residues and *β*-D-glucopyranosyl residues	Liang et al. (2018)
CP	Flowers of *Chrysanthemum morifolium*	NA	NA	NA	Tao et al. (2017, 2018)
PLS	Fruits of *Morinda citrifolia*	GalA, Gal, Ara, Rha, and Man in a ratio of 29.10:30.90:31.00:5.40:3.60	NA	NA	Batista et al. (2020)
WJPs	Sarcocarps of *Ziziphus jujuba*	Glc, Ara, GalA, and Gal in a ratio of 38.59:23.16:17.64:10.44	NA	NA	Yue et al. (2015)
ALP-1	Roots of *Arctium lappa*	Fru and Glc	5.12 × 10^3^	(2→1)-β-D-fructofuranose	Wang Y. et al. (2019)
MSP	Aerial parts of *Malva sylvestris*	Gal, Glc, UA, Ara, and Rha in a ratio of 4.00:5.00:14.00:6.00:1.00	1.30 × 10^6^	NA	Hamedi et al. (2016)
HMFO	Roots of *Cynanchum wilfordii*	Glc, Ara, Gal, Rha, and GalA	0.12–5.20 × 10^5^	NA	Cho et al. (2017)
PP	Rootstocks of *Rauvolfia verticillata*	GalA, Gal, Ara, Rha, Glc, and Man in a ratio of 51.00:6.80:7.10:2.60:31.30:0.70	0.50–3.00 × 10^5^	NA	Miao et al. (2019)
POLP	Aerial parts of *Portulacae oleracea*	NA	NA	NA	Wang et al. (2018), Wang Z. et al. (2020)
OP	Fruits of *Vaccinium oxycoccos*	GalA, Rha, Ara, Glc, and Gal in a ratio of 82.00:1.50:8.00:5.00:3.00	1.00–3.00 × 10^5^	NA	Popov et al. (2006)
ASPP	Tubers of *Ipomoea batatas*	Rha, Ara, Xyl, Man, and Glc in a ratio of 2.80:1.90:1.00:7.60:53.30	1.80 × 10^5^	1,4-linked Glc*p*	Sun et al. (2020)
MAP	Fruits of *Malus/ domestica*	GalA and Gal	0.50–1.00 × 10^4^	NA	Li et al. (2017)
**Polysaccharides from edible mushrooms**					
GLP	*Ganoderma lucidum*	NA	1.03 × 10^5^	1,6-lnked *β*-D-Glc*p*	Xie et al. (2019)
HECP	*Hericium erinaceus*	Glc, Gal, Ara, Xyl, Rha, and Man in a ratio of 76.71:14.26:4.04:2.57:1.32:1.14	8.67 × 10^4^	NA	Ren et al. (2018)
EP-1	*Hericium erinaceus*	Glc, Man, Gal	3.10 × 10^3^	(1→3)-linked glucan	Wang D. D. et al. (2019)
CMP33	*Poria cocos*	NA	15.23 × 10^4^	(1→3)-linked glucose	Liu X. F. et al. (2018)
IOP	*Inonotus obliquus*	Man, Rha, Glc, Gal, Xyl, and Ara in a ratio of 9.20:4.40:46.60:11.50:11.10:4.30	NA	NA	Chen et al. (2019)
DIP	*Dictyophora indusiata*	Glc	5.36 × 10^5^	β-(1→3)-D-glucan	Wang Y. L. et al. (2019)
PPS	*Pycnoporus sanguineus*	Glc, Man, and Gal	3.29 × 10^4^	NA	Li M. X. et al. (2020)
FVP	*Flammuliana velutipes*	Glc, Man, and Gal in a ratio of 56.20:29.70:14.10	5.48 × 10^4^	NA	Zhang et al. (2020)
LEP	*Lachnum* YM130	Man and Gal in a ratio of 3.80:1.00	1.31 × 10^6^	NA	Zong et al. (2020)
**Polysaccharides from seaweeds**					
GBP	*Gracilaria birdiae*	Gal and AnGal	0.38–2.60 × 10^6^	→4–3,6–anhydro–α–L–Gal*p* (1→3) β–D–Gal*p* 1→ segments	Brito et al. (2014)
ULP	*Ulva lactuca*	Rha, Xyl, Glc, and UA	NA	NA	Zhu et al. (2017)
ECP	*Eucheuma cottonii*	Gal	NA	NA	Sudirman et al. (2018)
BMP	*Blidingia minima*	Rha, Xyl, GluA, and Glc	NA	NA	Song et al. (2019)

**TABLE 3:** Summary of the mechanisms of polysaccharides from natural sources in the treatment of IBD.

Name	Source	Model	Mechanism of action	Reference
**Polysaccharides from edible and medicinal plants**				
APS	*Astragalus membranaceus*	DSS-induced C57BL/6 mice	Downregulation: TNF-α, IL-1β, IL-6, MPO, and NF-κB p-p65	Lv et al. (2017)
SP1-1	*Scutellaria baicalensis*	DSS-induced C57BL/6 mice; LPS-stimulated THP-1 cells	Upregulation: p65 (cytoplasm) and p-p65 (cytoplasm); downregulation: MPO, p-IKKα, -IKKβ, p-IκBα, p65 (nucleus), p-p65 (nucleus), NLRP3, caspase-1, cleaved caspase-1, IL-1β, pro-IL-1β, IL-18, and pro-IL-18	Cui et al. (2019)
ASP	*Angelica sinensis*	DSS-induced BALB/c mice; LPS-stimulated Caco-2 cells	Upregulation: ZO-1, occludin, claudin-1, and Bcl-2; downregulation: MPO, IL-6, IL-1β, TNF-α, Bax, and caspase-3	Cheng et al. (2020)
DOPS	*Dendrobium officinale*	DSS-induced BALB/c mice; LPS-stimulated NCM460 cells	Upregulation: IL-10; downregulation: IL-1β, IL-6, IL-18, TNF-α, IFN-γ, NLRP3, ASC, caspase1, and *β*-arrestin1	Liang et al. (2018)
CP	*Chrysanthemum morifolium*	TNBS-induced SD rats	Upregulation: IL-4, IL-10, IL-13, SOD, *Firmicutes*/*Bacteroidetes*, *Butyricicoccus*, *Clostridium*, *Lactobacillus*, *Bifidobacterium*, *Lachnospiraceae,* and *Rikenellaceae*; downregulation: MPO, TNF-α, IL-1β, IL-6, IF-17, IL-23, IFN-γ, MDA, TLR4, p65, p-p65, STAT3, p-STAT3, JAK2, *Escherichia, Enterococcus*, and *Prevotella*	Tao et al. (2017, 2018)
PLS	*Morinda citrifolia*	HOAc-induced Swiss mice	Upregulation: GSH; downregulation: MPO, MDA, NO_3_/NO_2_, and COX-2	Batista et al. (2020)
WJPs	*Ziziphus jujuba*	TNBS-induced SD rats; TNF-α-stimulated Caco-2 cells	Upregulation: occludin, claudin-1, claudin-4, ZO-1, p-AMPK, and p-ACC; downregulation: TNF-α, IL-1β, IL-6, and MPO	Yue et al. (2015)
ALP-1	*Arctium lappa*	DSS-induced ICR mice	Upregulation: IL-10, IgA, *Firmicutes, Ruminococcaceae, Lachnospiraceae, and Lactobacillus*; downregulation: IL-1β, IL-6, TNF-α, *Proteobacteria*, *Alcaligenaceae*, *Staphylococcus*, and *Bacteroidetes*	Wang Y. et al. (2019)
HMFO	*Cynanchum wilfordii*	DSS-induced BALB/c mice; LPS-stimulated RAW264.7 cells	Downregulation: MPO, PGE_2_, NO, TNF-α, IL-6, iNOS, COX-2, p-p65, p-IκBα, p-IKK α/β, p-JNK, p-ERK, and p-p38	Cho et al. (2017)
PP	*Rauvolfia verticillata*	DSS-induced BALB/c mice	Upregulation: IκBα; downregulation: MPO, TNF-α, IL-6, p65, ERK, JNK, and p38	Miao et al. (2019)
POLP	*Portulacae oleracea*	DSS-induced Kunming mice	Upregulation: IL-10, IκBα, and NF-κB p65 (cytoplasm); downregulation: TNF-α, IL-1β, IL-6, IL-18, PGE_2_, Bcl-2, survivin, p-STAT3, COX-2, and NF-κB p65 (nucleus)	Wang et al. (2018), Wang Z. et al. (2020)
OP	*Vaccinium oxycoccos*	HOAc-induced A/HeJ mice	Downregulation: MDA	Popov et al. (2006)
ASPP	*Ipomoea batatas*	DSS-induced ICR mice	Upregulation: AA, PA, BA, and *Firmicutes*; downregulation: TNF-α, IL-1β, IL-6, *Bacteroidetes*, *Proteobacteria*, and *Actinobacteria*	Sun et al. (2020)
MAP	*Malus domestica*	DSS-induced ICR mice	Upregulation: IL-22BP; downregulation: IL-22, p-STAT3, Bcl-2, and cyclin D1	Li et al. (2017)
**Polysaccharides from edible mushrooms**				
GLP	*Ganoderma lucidum*	DSS-induced Wistar rats	Upregulation: TA, AA, PA, BA, *Ruminococcus_1*, *Ccl5, Cd3e, Cd8a, Il21r, Lck, and Trbv*; downregulation: *Escherichia–Shigella*, *Ccl3, Gro, Il11, Mhc2, and Ptgs*	Xie et al. (2019)
HECP	*Hericium erinaceus*	DSS-induced C57BL/6 mice	Upregulation: T-SOD and *Bacteroidetes*; downregulation: NO, MDA, MPO, IL-6, IL-1β, TNF-α, COX-2, iNOS, p-p65/p65, p-IκBα/IκBα, pAkt/Akt, p-p38/p38, p-ERK/ERK, p-JNK/JNK, *Verrucomicrobia*, *Actinobacteria*, *Arthrobacter* spp., *Methylibium* sp., *Succinivibrio* sp., *Desulfovibrio* sp., and *Akkermansia muciniphila*	Ren et al. (2018)
EP-1	*Hericium erinaceus*	HOAc-induced SD rats H_2_O_2_-induced Caco-2 cells	Upregulation: MMP, SOD, OCR, ATP, Bcl-2, AA, PA, BA, VA, IBA, IVA, GPR41, GPR43, and IgM; downregulation: MDA, TNF-α, IL-1, IL-6, ROS, p-p65, p65, caspase-3, and C3	Shao et al. (2019); Wang D. D. et al. (2019)
		
CMP33	*Poria cocos*	TNBS-induced Kunming mice	Upregulation: IL-4, IL-10, and DHT; downregulation: MPO, MDA, TNF-α, IL-6, L-1β, IL-12, IFN-γ, IL-2, IL-17, Hmgcs2, Fabp2, Hp, B4galnt2, B3gnt6, Sap, Ca1, and oleic acid	Liu X. F. et al. (2018)
IOP	*Inonotus obliquus*	DSS-induced BALB/c mice	Upregulation: ZO-1, occludin, IL-4, IL-10, GATA-3, Foxp3, and p-STAT6; downregulation: p-STAT1, p-STAT3, IFN-γ, IL-17, T-bet, and ROR-γt	Chen et al. (2019)
DIP	*Dictyophora indusiata*	DSS-induced C57BL/6 mice	Upregulation: GSH, HO-1, IL-10, Bcl-2, TJP1, and IRF4; downregulation: MDA, MPO, TNF-α, IL-6, IL-1β, IL-18, NLRP3, p-IκBα, p-STAT3, Bax, IRF5, and CD86	Wang Y. L. et al. (2019)
PPS	*Pycnoporus sanguineus*	DSS-induced BALB/c mice	Upregulation: ZO-1, E-cadherin, PCNA, Th1, MCP-1β, ULK1, LC3 I, p62, Beclin-1, and LC3 II; downregulation: LPS, Proportions of Th cells, Th2, Th17, Treg, MPO, IL-10, IL-12p40, IL-15, IL-17, and LC3 II/I	Li M. X. et al. (2020)
FVP	*Flammuliana velutipes*	DSS-induced SD rats	Upregulation: SOD, AA, PA, BA, IVA, and VA; downregulation: MPO, NO, DAO, TLR4, NF-κB, and p-p65	Zhang et al. (2020)
LEP	*Lachnum* YM130	DSS-induced ICR mice	Upregulation: ZO-1, occludin, claudin-1, E-cadherin, claudin-3, claudin-7, MUC1, MUC2, TFF3, Relmβ, Reg3β, Reg3γ, PPARγ, CAT, SOD, T-AOC, IRE1α, XBP1; downregulation: TNF-α, IL-1β, IL-6, iNOS, COX-2; NO, PGE_2_; NF-κB p-p65, p-IκBα, p-STAT3 MPO, Ly6G; CD4^+^IL-10^+^, F4/80, NLRP3, ASC, caspase-1, Bip, ATF6, PERK, CHOP, C-Cas3, MDA, nitrotyrosine, and NOx	Zong et al. (2020)
**Polysaccharides from seaweeds**				
GBP	*Gracilaria birdiae*	TNBS-induced Wistar rats	Upregulation: GSH; downregulation: IL-1β, TNF-α, MPO, MDA, and NO_3_/NO_2_	Brito et al. (2014)
ULP	*Ulva lactuca*	DSS-induced C57BL/6 mice	Upregulation: GSH, GPx, and Se; downregulation: MPO, IL-6, TNF-α, iNOS, COX-2, CD68, NF-κB p-p65, and p-IκBα	Zhu et al. (2017)
ECP	*Eucheuma cottonii*	DSS-induced BALB/c mice	Upregulation: IL-10; downregulation: TNF-α, IL-1β, and IL-6	Sudirman et al. (2018)
BMP	*Blidingia minima*	DSS-induced C57BL/6J mice and IPEC-J2 cells	Upregulation: ZO-1, occludin, claudin-1, IL-10, and DAO; downregulation: MPO, EPO, ET-1, TNF-α, IL-1β, p-NF-κB, p-IκBα, and p-AKT	Song et al. (2019)

### Astragalus membranaceus

*A. membranaceus* belongs to the Leguminosae family and is one of the most commonly used Chinese herb medicine in China. It has many pharmacological effects, such as immune regulation, antitumor, antivirus, and so on (Fu et al., 2014). Polysaccharides, triterpenoids, and flavonoids are the three main active components of *A. membranaceus* (Jin et al., 2014). Lv et al. (2017) prepared a polysaccharide APS from *A. membranaceus* and evaluated the therapeutic roles of APS and delineated the possible molecular mechanisms in the DSS-induced mouse colitis model. The results demonstrated that APS treatment observably improved colitis-related clinical signs and pathological damage of colon caused by DSS exposure. Moreover, APS could also significantly inhibit the intestinal inflammation response through downregulating the colonic mRNA expression and release of pro-inflammatory mediators, including TNF-α, IL-1β, and IL-6, and reducing the MPO activity. In addition, APS treatment was observed to block NF-κB activation by suppressing the phosphorylation of NF-κB p65. Overall, these found implied that APS could serve as a natural therapeutic approach for the treatment of IBD.

### Scutellaria baicalensis

*S. baicalensis* is a species of the flowering plant in the *Lamiaceae* family. Its dried roots have been used for over 2,000 years as a traditional Chinese medicine known as *Huang*-*Qin* (Zhao et al., 2016). Cui et al. (2019) obtained a purified polysaccharide SP1-1 from *S. baicalensis*, and found that SP1-1 administration can effectively improve DSS-induced colitis by reducing DAI scores, colonic lesions, and MPO activity. SP1-1 significantly suppressed the production of pro-inflammatory cytokines TNF-α, IL-1β, and IL-18 in the serum and colon of DSS-induced colitis mice and LPS-induced THP-1 derived macrophages. In addition, SP1-1 remarkably decreased the colonic CD11b^+^ macrophage infiltration. Meanwhile, SP1-1 treatment effectively inhibited the activation of the NF-κB pathway through reducing phosphorylation of IKKα, IKKβ, and IκBα, and inhibiting the translocation of NF-κB p65 from cytoplasm to nucleus. Furthermore, the elevated expression of NLRP3, caspase-1, cleaved caspase-1, IL-1β, pro-IL-1β, IL-18, and pro-IL-18 in DSS treated mice was mitigated by SP1-1, resulting in the inactivation of NLRP3 inflammasome. These data implied that the anti-inflammatory effect of SP1-1 against DSS-induced colitis was closely related to its inhibition of NLRP3 inflammasome and NF-κB signaling pathways. SP1-1 may be served as a novel candidate drug to treat IBD in future.

### Angelica sinensis

**FIGURE 3:**
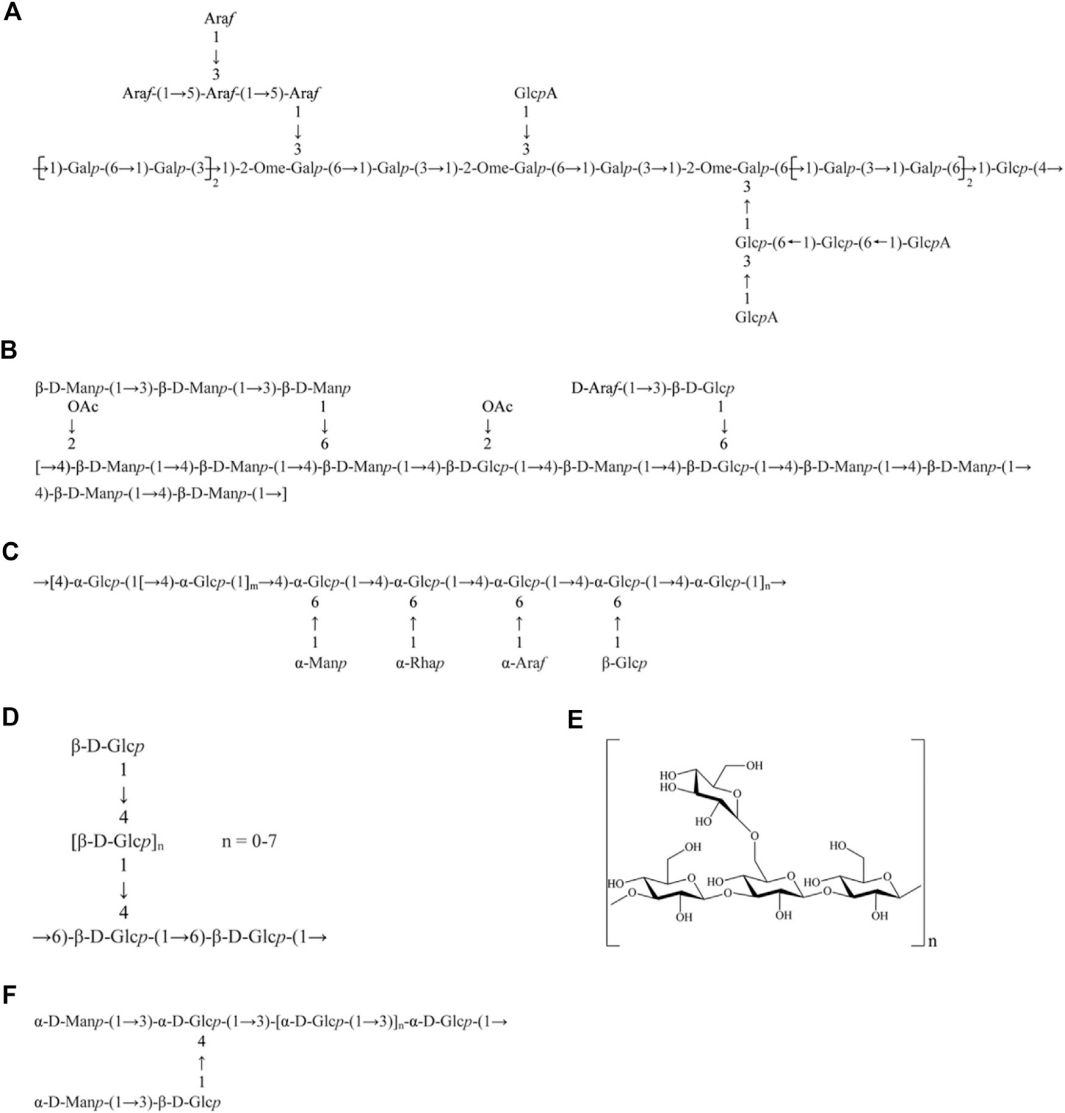
Chemical structures of polysaccharides from natural sources for the treatment of IBD. **(A)** ASP from *Angelica sinensis*; **(B)** DOPS from *Dendrobium officinale*; **(C)** ASPP from *Ipomoea batatas*; **(D)** GLP from *Ganoderma lucidum*; **(E)** DIP from *Dictyophora indusiata*; and **(F)** EP-1 from *Hericium erinaceus*.

*A. sinensis* belongs to the Umbelliferae family, which has been traditionally used in Chinese medicinal formulation for a long time and is also commonly used as a dietary supplement in Europe and America (Wei et al., 2016). Polysaccharides are a class of phytochemicals in *A. sinensis*, which have been proved to have many pharmacological activities (Jin et al., 2012). Cheng et al. (2020) extracted an acidic polysaccharide ASP (Figure 3A) from *A. sinensis* and investigated the protective effects of ASP on DSS-induced colitis. Results showed that ASP observably attenuate the severity of colitis symptoms manifested as the reduction of weight loss, DAI score, and colon length shortening induced by DSS. Furthermore, the mRNA expressions of pro-inflammatory cytokines (TNF-α, IL-6, and IL-1β) and MPO activity caused by DSS were strikingly inhibited by ASP treatment. ASP also improved the colonic barrier function *via* enhancing the expressions of TJs proteins (ZO-1, occludin, and claudin-1) and decreasing cell membrane permeability in LPS-stimulated Caco-2 cell. In addition, ASP could significantly mitigate intestinal epithelium cell apoptosis and promote proliferation through upregulating Bcl-2 protein expression and downregulating Bax and caspase-3 levels in colon tissues of DSS-induced colitis mice. Collectively, these results manifested that ASP can be a potential natural ingredient for the treatment of IBD.

### Dendrobium officinale

*D. officinale*, a precious plant of the Orchidaceae family, possesses extremely high medicinal and edible values. It has been broadly applied to treat gastrointestinal diseases in China for thousand years, which was the earliest recorded in Shennong's Classic of Materia Medica (Tang et al., 2017). Liang et al. (2018) obtained a heteropolysaccharide DOPS (Figure 3B) from *D. officinale*, and found DOPS could obviously ameliorate the clinical symptoms, reduce mortality, and relieve pathological damage of colon in colitis mice induced by DSS. Interestingly, DOPS treatment pronouncedly regulated the imbalance of pro-/anti-inflammatory mediators through reducing the production of pro-inflammatory cytokines (TNF-α, IL-6, IL-1β, IL-18, and IFN-γ) and augmenting the anti-inflammatory IL-10 level, and thus descending the ratio of pro-/anti-inflammatory cytokines. In addition, DOPS could significantly inhibit the expression of NLRP3, ASC, caspase1, and *β*-arrestin1 in DSS-induced colitis mice and LPS-stimulated NCM460 cells. In summary, these data indicated that DOPS administration exhibited a therapeutic effect on DSS-induced experimental colitis in mice, which was probably associated with the suppression of NLRP3 inflammasome activation and *β*-arrestin1 signaling pathway, and the subsequent expression of pro-inflammatory cytokines. DOPS is expected to be a potential candidate component for the treatment of inflammatory diseases like IBD in the future.

### Chrysanthemum morifolium

*C. morifolium*, a medicinal and edible plant of the Asteraceae family, has been broadly applied in clinical practice for thousands of years in China and Korea (Tao et al., 2016). Tao et al. (2017), Tao et al., (2018) abstracted polysaccharides from *C. morifolium* and found that CP possessed pronouncedly protective effects on TNBS-induced colitis in rats. CP administration could significantly enhance the production of anti-inflammatory mediators IL-4, IL-10, and IL-13 while decreased the secretion of pro-inflammatory factors, including TNF-α, IL-1β, IL-6, IL-17, IL-23, and IFN-γ. Moreover, CP inhibited oxidative stress by increasing the SOD level and reducing the MDA content. Further mechanistic study illustrated that the anti-inflammatory roles of CP might be related to blocking the activation of NF-κB/TLR4 and IL-6/JAK2/STAT3 signaling pathways by inhibiting the mRNA levels of NF-κB, TLR4, IL-6, JAK2, and STAT3, and the protein expression of NF-κB p65, p-p65, TLR4, JAK2, STAT3, and p-STAT3 in colonic tissues of TNBS-induced colitis rats. Additionally, 16S rRNA sequencing analysis result revealed that CP remarkably raised the ratio of *Firmicutes*/*Bacteroidetes*, and elevated microbial diversity and community richness in rats with colitis. The relative abundance of pathogens (*Escherichia, Enterococcus,* and *Prevotella*) was downregulated, while the levels of probiotics including *Butyricicoccus*, *Clostridium*, *Lactobacillus*, *Bifidobacterium*, *Lachnospiraceae,* and *Rikenellaceae* were increased after CP treatment. Correlation analysis demonstrated that the gut microflora was correlated closely with the expression of cytokines, and they interacted with each other to modulate immune function. In conclusion, CP could alleviate IBD by regulating intestinal microecological balance and maintaining immune homeostasis, which promote the curative drug development of IBD.

### Morinda citrifolia

*M. citrifolia*, commonly known as noni, belongs to the Rubiaceae family and is a native tropical shrub in Southeast Asia. It has been used in traditional medicine for more than 2,000 years. Nowadays, *M. citrifolia* attracted the attention of researchers from the pharmaceutical and food industry for different therapeutic purposes (Almeida et al., 2019). Batista et al. (2020) extracted polysaccharides (PLS) from the fruits of *M. citrifolia*. PLS was found to ameliorate the intestinal damage and MPO activity in acetic acid–induced colitis. PLS significantly reduced oxidative damage by increasing the GSH level and decreasing the content of MDA and NO_3_/NO_2_. Moreover, PLS could prominently inhibit the expression of TNF-α, IL-1β, and COX-2. This result demonstrated that PLS exhibited an anti-inflammatory effect against colitis by mitigating inflammatory response and oxidative stress in the inflamed colon, suggesting PLS possessed therapeutic potential against inflammatory diseases like IBD.

### Ziziphus jujuba

*Z. jujuba*, also called jujube, is a thorny, rhamnaceous deciduous plant broadly distributed in northern China. Its fruits are much admired for their high nutritional and medicinal values (Gao et al., 2013). Yue et al. (2015) abstracted a crude polysaccharide WJPs from wild jujube sarcocarp and investigated the therapeutic roles of WJPs on TNBS-induced colitis rats and TNF-α-stimulated Caco-2 cells. Results showed that WJPs could observably ameliorate the severity of colitis and attenuate mucosal injury in colitis rats. In addition, WJPs mitigated the intestinal inflammatory response by inhibiting the levels of TNF-α, IL-1β, IL-6, and MPO activity. Furthermore, WJPs was found to improve the intestinal epithelial barrier function as WJPs could significantly reverse TNF-α-induced increase of FD-4 flux and decrease of TER in Caco-2 cells, and upregulate the expression of TJs proteins (ZO-1, occludin, claudin-1, and claudin-4) in colonic tissues of rats with TNBS-induced colitis. Further mechanistic study revealed that WJPs remarkably induced activation of the AMPK pathway through upregulation of the phosphorylation of AMPK and ACC *in vivo* and *in vitro*. Taken together, WJPs possess prominently protective effect on colitis, at least partly through enhancing intestinal barrier function, and suppressing inflammation response. WJPs may be used as a promising candidate to meet the medication needs of IBD.

### Arctium lappa

*A. lappa*, also called burdock, is a medicinal and edible plant belonging to the Asteraceae family. It is mainly distributed in China and Western Europe and traditionally used to treat various diseases. Nowadays, *A. lappa* is regarded as a valued source for secondary metabolites, which exhibits many biological activities and pharmacological functions (Gao et al., 2018). Wang Y. et al. (2019) extracted a water-soluble polysaccharide ALP-1 from *A. lappa* and applied the DSS-induced mice model to assess the protective effect of ALP-1 on colitis. The results revealed that ALP-1 could dramatically increase anti-inflammatory cytokine IL-10 and alleviate pro-inflammatory mediator (IL-1β, IL-6, and TNF-α) levels in colon and serum of colitis mice. Moreover, the reduced level of IgA caused by DSS exposure was notably increased after ALP-1 treatment. In addition, compared with the DSS-induced colitis group, ALP-1 treatment remarkably enhanced the relative abundance of probiotics, including *Firmicutes*, *Ruminococcaceae*, *Lachnospiraceae,* and *Lactobacillus*, while descended the levels of pathogen, such as *Proteobacteria*, *Alcaligenaceae*, *Staphylococcus,* and *Bacteroidetes*. In conclusion, the research showed that the anti-colitis effect of ALP-1 may be associated with the regulation of gut microbiota structure and the imbalance of inflammatory cytokines. ALP-1 may be applied as a dietary supplement agent for patients with IBD.

### Malva sylvestris

*M. sylvestris*, known as common mallow, belongs to the Malvaceae family. It is a medicinal and edible plant that mainly grows in Europe, North Africa, and Asia. Studies have proved that *M. sylvestris* possess beneficial gastrointestinal effects (Gasparetto et al., 2012). Polysaccharides are reported to be the major active components of *M. sylvestris*. Hamedi et al. (2016) found that the isolated polysaccharide MSP from *M. sylvestris* could effectively ameliorate macroscopic and microscopic parameters and inhibit inflammation symptoms of colitis, which exhibited obviously a protective effect against IBD induced by acetic acid in rats.

### Cynanchum wilfordii

*C. wilfordii* belongs to the Asclepiadaceae family and is mainly distributed in Korea, Japan, and China. Its underground roots are traditionally used for the treatment of gastrointestinal diseases (Jiang et al., 2011). Cho et al. (2017) prepared a crude polysaccharide HMFO from *C. wilfordii*, and explored the anti-inflammatory effects and underlying mechanisms of HMFO in DSS-induced mouse colitis and LPS-stimulated RAW 264.7 cells. Results showed that HMFO dramatically relieved the pathological characteristics and histological damage of colitis, reduced MPO activity, and inhibited the production of pro-inflammatory factors TNF-α and IL-6 in the colon of colitis mice. Furthermore, HMFO significantly downregulated the expression of iNOS, COX-2, and phosphorylated NF-κB in mice. *In vitro*, HMFO sharply mitigated several cytokines and enzymes associated with inflammation, including NO, PGE_2_, TNF-α, IL-6, iNOS, and COX-2. Further mechanism study revealed that HMFO blocked the activation of NF-κB and MAPK signaling pathways by retarding the phosphorylation of NF-κB p65, IκBα, IKK α/β, p38, ERK, and JNK. These results speculated that HMFO is a promising remedy to treat IBD.

### Rauvolfia verticillata

*R. verticillata* belongs to the Apocynaceae family and is mainly distributed in China and Vietnam. Its roots and leaves are used for medicinal purposes including treating hypertension, hyperthermia, insomnia, and so on (Hong et al., 2012). Miao et al. (2019) obtained a pectic polysaccharide PP from the rootstocks of *R. verticillata* and found that PP treatment attenuated the overall physical activity, DAI, and morphological damage in DSS-treated colitis mice. Meanwhile, PP dramatically reduced TNF-α, IL-6, and MPO activity. Furthermore, the down-regulated IκBα level and up-regulated expression of NF-κB p65, ERK, JNK, and p38 caused by DSS exposure were reversed by PP treatment. In short, these findings strongly suggested that PP can inhibit the colonic inflammation *via* suppressing NF-κB and MAPK pathways, providing a scientific basis for the application of PP as an effective therapeutic agent for the treatment of IBD.

### Portulacae oleracea

*P. oleracea* belongs to the Portulacaceae family and is a widespread edible and medicinal plant for alleviating various diseases (Iranshahy et al., 2017). Wang et al. (2018), Wang Z. et al. (2020) acquired a polysaccharide POLP from *P. oleracea* and found that POLP could relieve DSS-induced clinical symptoms and improve histopathological damage. The levels of pro-inflammatory factors TNF-α, IL-1β, IL-6, IL-18, and PGE_2_ were markedly decreased, whereas the secretion of anti-inflammatory mediator IL-10 was increased after POL administration. Moreover, POLP sharply upregulated the IκBα protein expression, inhibited the translocation of NF-κB p65 from cytoplasm to nucleus, and downregulated the NF-κB p65 related proteins (including Bcl-2 and survivin) in DSS-treated mice. In addition, POLP significantly suppressed the COX-2 protein expression and the phosphorylation of STAT3. Thus, it was speculated that POLP exhibited excellent protective effects on IBD, and the mechanisms were closely associated with the inhibition of NF-κB and STAT3/COX-2 pathways.

### Vaccinium oxycoccos

*V. oxycoccos*, also called European cranberry, is a plant belongs to the Ericaceae family. Its fruits are a valuable source of antioxidants and other biologically active substances (Jurikova et al., 2019). Popov et al. (2006) extracted a pectic polysaccharide OP from *V. oxycoccos* and found that the colonic macroscopic damage score and total injury area were obviously relieved in the OP treatment group compared with those of the 5% acetic acid–induced colitis group. OP has shown to reduce the MPO activity in colon tissue and increase the mucus content. Moreover, OP pretreatment can significantly reduce the colon MDA level. OP has also been found to suppress inflammation, which is estimated by preventing vascular permeability. In addition, the adhesion of peritoneal neutrophils and macrophages was mitigated after OP administration. Overall, OP has a therapeutic effect on acetic acid–induced colitis in mice. The protective effect of OP may be related to the inhibition of neutrophil infiltration and antioxidant.

### Ipomoea batatas

*I. batatas*, a plant belongs to the Convolvulaceae family, is generally called purple sweet potato for purple flesh and treated as a nutritionally rich food (Mohanraj and Sivasankar, 2014). Sun et al. (2020) abstracted a novel alkali-soluble polysaccharide ASPP (Figure 3C) from *I. batatas*, and assessed the protective effects of ASPP on DSS-induced colitis in mice. The results demonstrated that ASPP improved the immune organ (spleen and thymus) indices and alleviated the colonic pathological damage. In addition, the levels of pro-inflammatory cytokines, including TNF-α, IL-1β, and IL-6 in colonic tissue and serum were significantly inhibited by ASPP treatment. ASPP could also elevate the SCFAs production, such as AA, PA, and BA in DSS-induced mice. Moreover, the 16S rRNA sequencing result suggested that ASPP regulated the compositions of gut microbiota in DSS-induced colitis mice by increasing the relative abundance of *Firmicutes* and reducing the levels of *Bacteroidetes*, *Proteobacteria*, and *Actinobacteria*. In conclusion, ASPP diminished intestinal inflammation through mitigating pro-inflammatory factors and modulating the gut microbiota structure of DSS-induced colitis mice.

### Malus pumila

*M. pumila* belongs to the Rosaceae family, and its fruits (namely apple) have very high nutritional values for containing abundant minerals and vitamins. Meanwhile, apples contain a variety of phytochemicals with bioactivity (Boyer and Liu, 2004). Li et al. (2017) extracted a polysaccharide MAP from *M. pumila* and applied a DSS-induced mouse colitis, MCA-38 cell line, and DC2.4 cell to investigate the anti-inflammatory properties and underlying mechanisms of MAP. Results showed that MAP significantly ameliorated intestinal toxicity caused by DSS in mice, and suppressed the proliferation of MCA-38 cells. Besides, MAP treatment remarkably increased IL-22BP levels, while inhibited the expression of IL-22, p-STAT3, Bcl-2, and cyclin D1 *in vivo* and *in vitro*. In short, these data indicated that MAP was likely to exert a protective effect on colitis *via* modulating the expression and function of IL-22 and IL-22BP, which provided a theoretical basis for apples used to prevent colitis.

## Polysaccharides From Mushrooms

Mushrooms are fungi that generally live on dead trees. According to statistics, there are more than 2000 kinds of edible mushrooms in the world, including about 700 kinds of mushrooms with medicinal value. Edible mushroom is one of the important sources of nutrients in people's daily life, and its factory cultivation is an emerging sunrise industry in the 21st century. Meanwhile, due to the richness of various active components, mushrooms are increasingly appreciated for their health and medicinal value (Roupas et al., 2012). There have been several polysaccharides from this source and have shown evident efficacy for IBD. Tables 2, 3 listed the chemical and pharmacological information of polysaccharides from mushrooms in the treatment of IBD, respectively.

### Ganoderma lucidum

*G. lucidum*, called *Ling-Zhi* in Chinese and Reishi in Japanese, is a famous medicinal mushroom growing on dead wood. Its main components are polysaccharides and triterpenes, which have various pharmacological activities, such as immunomodulation, antitumor, and anti-oxidation (Ahmad, 2018; Lu et al., 2020). Xie et al. (2019) reported that a polysaccharide GLP (Figure 3D) from *G. lucidum* remarkably reduced DSS-induced DAI scores and augmented SCFAs levels (including TA, AA, PA, and BA). Moreover, GLP effectively regulated the intestinal microbiota structure by increasing the amount of prebiotics (e.g., *Ruminococcus_1*) and reducing some pathogens (e.g., *Escherichia–Shigella*). Transcriptional analysis indicated that GLP regulated the expression of genes enriched in inflammation-related KEGG pathways, such as elevating *Ccl5*, *Cd3e*, *Cd8a*, *Il21r*, *Lck,* and *Trbv*, and decreasing the levels of *Ccl3*, *Gro, Il11*, *Mhc2,* and *Ptgs2*, resulting in improvement of immunity and reduction of inflammation and colonic carcinoma risk. Therefore, these results demonstrated that GLP ameliorated DSS-induced colitis and may have potential application prospects in the remission of IBD.

### Hericium erinaceus

*H. erinaceus*, a kind of edible and medicinal mushroom, is commonly used to prevent and treat gastrointestinal disorders (Friedman, 2015). Ren et al. (2018) isolated a polysaccharide HECP from *H. erinaceus*, and found that HECP obviously improved DSS-induced clinical symptoms and pathological damage. HECP ameliorated oxidative damage through inhibiting the levels of NO, MDA, and MPO, and increasing the T-SOD activity. HECP could also suppress the development of inflammation *via* inhibiting COX-2, iNOS, and phosphorylation of NF-κB (p65 and IκBα), MAPK (p38, ERK, and JNK), and PI3K/AKT (AKT) signaling pathways in colon tissues of DSS-induced colitis mice. As a consequence, the mRNA expression and levels of pro-inflammatory cytokines, including TNF-α, IL-6, and IL-1β, were dramatically suppressed after HECP treatment. Moreover, HECP could regulate gut microbiota dysbiosis, manifested as the reduction of *Verrucomicrobia* and *Actinobacteria*, elevation of *Bacteroidetes*, and subsequently the decrease of some bacterial species, including *Arthrobacter* spp., *Methylibium* sp., *Succinivibrio* sp., and *Akkermansia muciniphila* and the enhancement of *Desulfovibrio* sp. in fecal sample, resulting in microbiota composition close to that of normal mice and prevent intestinal barrier damage caused by DSS. Taken together, HECP exhibited an important protective effect on DSS-induced colitis in mice, at least partly through the modulation of oxidative stress, inflammation-related cytokines and signaling pathways, and gut microbiota dysbiosis, indicating HECP can serve as a potential dietary nutrient against IBD in the future.

Another purified polysaccharide EP-1 (Figure 3F) was extracted from *H. erinaceus*, and research showed that EP-1 observably relieved the clinical symptoms and colonic mucosa damage in acetic acid–induced colitis rats. EP-1 effectively regulated the gut microbial structure, increased the levels of SCFAs (including AA, PA, BA, VA, IBA, and IVA), GPR41, and GPR43. EP-1 treatment could also upregulate SOD activity, downregulate MDA and ROS production, and thus inhibit oxidative damage both in acetic acid–induced colitis mice and in H_2_O_2_-induced Caco-2 cells. Consequently, the mitochondria integrity and function were remarkably improved, indicated by the elevated levels of MMP, OCR, and ATP. Furthermore, EP-1 was found to exhibit an appreciable anti-inflammatory effect, which was closely associated with the blockade of NF-κB pathways by decreasing the expression of NF-κB p-65 and p-p65 in colonic tissue and subsequently reducing the production of pro-inflammatory cytokines TNF-α, IL-1, and IL-6 in rat serum. EP-1 also exerted an immunoregulation effect through increasing the IgM content and decreasing the C3 level. Additionally, EP-1 treatment inhibited intestinal epithelial cells apoptosis by increased Bcl-2 and decreasing caspase-3 activation (Shao et al., 2019; Wang D. D. et al., 2019). Overall, these results proved that EP-1 shows potential for the development of novel functional foods and drugs to treat IBD.

### Poria cocos

*P. cocos*, an edible and pharmaceutical fungus, is widely applied in the formulation of tea supplements, cosmetics, and functional foods (Rios, 2011). Polysaccharides are the major component of *P. cocos*, which have aroused wide attention on the structural features and pharmacological activities (Jia et al., 2016). A novel polysaccharide CMP33 was abstracted from *P. cocos*. Animal experiment found that after the administration of CMP33, the elevated mortality rate, DAI scores, MPO activity, and macro- or microscopic histopathological score induced by TNBS in mice were remarkably mitigated (Liu X. F. et al., 2018). Furthermore, CMP33 significantly reduced the release of pro-inflammatory cytokines (TNF-α, IL-6, L-1β, IL-12, IFN-γ, IL-2, and IL-17), whereas triggered anti-inflammatory factors IL-4 and IL-10 in the colon tissue and serum of colitis mice. Besides, CMP33 could effectually abrogate lipid peroxidation by decreasing MDA content in TNBS-induced colitis mice. iTRAQ-based proteomics revealed that CMP33 exhibited anti-inflammation effect by decreasing seven proteins (B3gnt6, B4galnt2, Ca1, Fabp2, Hmgcs2, Hp, and Sap). Additionally, GC-TOF-MS-based metabolomics data suggested that oleic acid and DHT might be the targets of CMP33. Collectively, these results indicated that CMP33 exhibited an important protective effect on colitis in mice.

### Inonotus obliquus

*I. obliquus*, the most promising medicinal fungus, grows on the birch trunks at low latitude in Europe and North America. Studies have shown that polysaccharides of *I. obliquus* may potentially be used for treating many diseases including tumors, diabetes, and colitis (Duru et al., 2019). *I. obliquus* polysaccharide (IOP) administration was reported to strikingly relieve DSS-induced chronic murine intestinal inflammatory symptoms (Chen et al., 2019). IOP treatment could significantly ameliorate DAI and the mucosa damage, where increase the TJs proteins ZO-1 and occludin in colon tissues. IOP could also upregulate the expression of Treg and Th2 and downregulate Th17 and Th1 in colon tissues, spleen, and mesenteric lymph nodes, resulting in reducing the mRNA levels of IFN-γ, IL-17, T-bet, and ROR-γt, and enhancing that of IL-4, IL-10, GATA-3, and Foxp3. Moreover, the elevated p-STAT1 and p-STAT3 and reduced p-STAT6 in DSS-induced mice were dramatically reversed by IOP. Taken together, these results demonstrated that IOP regulated the balance of Th1/Th2 and Th17/Treg through modulating the JAK-STAT pathway in DSS-induced colitis mice, suggesting IOP as a potential natural effective therapeutic agent or options for IBD.

### Dictyophora indusiata

*D. indusiata*, a saprophytic fungus of bamboo forest, has been one of the most popular edible mushrooms for its edible values and medicinal functions (Habtemariam, 2019). Wang Y. L. et al. (2019) extracted a purified polysaccharide DIP (Figure 3E) from *D. indusiata*, and investigated the anti-colitis effect and underlying mechanism of DIP in DSS-treated mice. Results showed that DIP could ameliorate colitis manifested as the reduction of clinical DAI scores, spleen index, and colonic histpathologic damage induced by DSS. Immunofluorescence staining illustrated that DIP significantly increased the colonic TJP1 protein expression, resulting in repairing epithelium barrier function. DIP could also relieve intestine oxidative stress by decreasing the MDA content and increasing the expression of GSH and HO-1 in the colonic tissue. Meanwhile, DIP treatment remarkably regulated inflammation responses through upregulating the anti-inflammatory cytokine IL-10 level and downregulating MPO activity and the release of pro-inflammatory cytokines TNF-α, IL-6, IL-1β, and IL-18 in DSS-induced colitis. DIP treatment was found to block the NF-κB, STAT3, and NLRP3 pathways *via* reducing the expression of NLRP3, p-IκBα, and p-STAT3, which may be responsible for the anti-inflammatory mechanism of DIP on colitis. Additionally, DIP could modulate macrophage subset through promoting DSS-induced IRF4 protein expression and inhibiting IRF5 and CD86 protein levels. Furthermore, DIP significantly inhibited DSS-induced apoptosis, which was associated with an upregulation of Bcl-2 expression and downregulation of Bax expression and TUNEL positive nuclei in colonic tissues. In conclusion, these results indicated that DIP exerted an obvious anti-colitis efficacy by reducing oxidative stress and inflammation, inhibiting key inflammatory signaling pathways, and regulating the macrophage subset. DIP could be served as a functional food or nutraceutical agent for the treatment of IBD.

### Pycnoporus sanguineus

*P. sanguineus* is a saprophytic fungus, which is widely applied in industry and medicine all over the world. Li M. X. et al. (2020) extracted a polysaccharide PPS from *P. sanguineus* and found that PPS could alleviate the colitis induced by DSS, which is manifested by reducing DAI, colon shortening, colonic MPO activity, and serum LPS concentration. PPS could effectively restored intestinal barrier function by increasing the expression of ZO-1, E-cadherin, and PCNA. Moreover, PPS significantly descended the proportions of Th cells and its subsets (Th2, Th17, and Treg), increased Th1 level, and suppressed the secretion of several inflammation-related cytokines, including IL-10, IL-15, IL-17, and IL-12p40 while increased chemokine MCP-1β. In addition, PPS mitigated the colitis-induced autophagy *via* downregulating the LC3 II/I ratio and upregulating the expression of ULK1, LC3 II, LC3 I, p62, and Beclin-1. This study revealed that PPS could relieve Th cell-associated inflammatory response, inhibit autophagy, and restore the intestinal barrier function, which might hold an important clinical implication for the treatment of IBD.

### Flammulina velutipes

*F. velutipes* is a well-known edible mushroom and widely consumed all over the world. Previous studies suggested that polysaccharides are the major component of *F. velutipes* and possess various bioactivities (Dong et al., 2020). Zhang et al. (2020) extracted a water-soluble polysaccharide FVP from *F. velutipes*, and found that FVP treatment could improve the DSS-induced colitis manifestations and epithelial mucous damage *in vivo*. The MPO and NO released by neutrophils in colonic inflammatory tissues were significantly inhibited by FVP. FVP also exhibited antioxidant capacity *via* decreasing the plasma DAO level, increasing intestinal SOD activity in DSS-induced colitis mice. Furthermore, FVP was found to show a favorable anti-inflammatory effect through downregulating the expression of TLR4, NF-κB, and NF-κB p-p65, resulting in suppression of the TLR4/NF-κB signaling pathway. Meanwhile, FVP could modulate the intestinal microbial dysbiosis and enhance the production of SCFAs (including AA, PA, BA, IVA, and VA). In summary, these results can promote the potential utilization of FVP as a functional food ingredient therapeutic for IBD.

### *Lachnum* YM130

*Lachnum* sp. is a genus of higher fungi with extremely high bioactivity value, which grows throughout Asia, America, and Europe. Zong et al. (2020) found that *Lachnum* YM130 polysaccharide (LEP) could remarkably alleviate clinical symptoms, colonic pathological damage, and inflammatory cell infiltration in DSS-induced colitis mice. Compared to the DSS-induced model group, the levels of MPO, Ly6G, F4/80 macrophage, and CD4^+^IL-10^+^ were significantly decreased in the LEP treatment group, suggesting LEP can inhibit the infiltration of inflammatory cells. LEP treatment also improved intestinal barrier integrity through increasing the expression of TJs proteins (including ZO-1, occludin, claudin-1, E-cadherin, claudin-3, and claudin-7), enhancing antimicrobial proteins expression Reg3β and Reg3γ, and upregulating the mRNA levels of mucus layer protective proteins, such as MUC1, MUC2, TFF3, and Relmβ. Moreover, LEP treatment strikingly inhibited the expression of inflammatory cytokines, including TNF-α, IL-1β, IL-6, NO, PGE_2_, iNOS, and COX-2 in colonic tissue of DSS-treated mice. Mechanistically, LEP treatment was found to block the activation of PPARγ/NF-κB and STAT3 pathways by upregulating PPARγ expression and retarding the phosphorylation of NF-κB p65, IκB, and STAT3 in DSS-induced colitis. LEP treatment also sharply downregulated the protein levels of NLRP3, ASC, caspase-1, and IL-1β in colonic tissues, thus inhibiting NLRP3 inflammasome activation. In addition, DSS-induced promotion of MDA, nitrotyrosine, and NOx levels and decrease of SOD, CAT, and T-AOC activities were significantly reversed after LEP administration. Meanwhile, the ER stress markers IRE1α and XBP1 were evidently elevated, whereas Bip, ATF6, PERK, CHOP, and C-Cas3 were descended in the LEP treatment group as compared with that in the DSS-induced colitis group. Overall, these results demonstrated that LEP possessed pronounced anti-colitis capacity and its mechanism might be tightly associated with protecting intestinal mucosal barrier function, suppressing inflammatory response, ER stress, and oxido-nitrosative stress.

## Polysaccharides From Seaweeds

Seaweed is an indispensable part of the marine ecosystem with many kinds, large quantity, and fast propagation. Moreover, it is also a rich and valuable resource for human beings, which plays an important role in functional food, food additives, marine drugs, organic fertilizer, bioenergy, and many other fields (Venkatesan et al., 2015). There are some studies on the treatment of IBD with polysaccharide from seaweeds, which have attracted extensive attention of researchers. Tables 2, 3 listed the chemical and pharmacological information of polysaccharides from seaweeds in the treatment of IBD, respectively.

### Gracilaria birdiae

*G. birdiae* is a kind of red algae, which is used to produce agar in Brazil with important economic value. In the past few years, the structure and bioactivity of *G. birdiae* polysaccharide have been widely studied (Souza et al., 2012). Brito et al. (2014) isolated an agar-type polysaccharide from *G. birdiae* and found that GBP treatment significantly ameliorated intestinal macroscopic and microscopic damage caused by TNBS in rats. In addition, it could also decrease the levels of pro-inflammatory cytokines IL-1β and TNF-α and MPO activity. Additionally, PLS relieved TNBS-caused oxidative stress injury manifested as the upregulation of the GSH level and reduction of MDA and NO_3_/NO_2_ concentration in the colonic mucosa. Together, this data concluded that GBP has a protective effect on intestinal epithelial damage by mitigating inflammatory cell infiltration, pro-inflammatory cytokine release, and oxidative stress.

### Ulva lactuca

*U. lactuca* is a kind of green giant algae, which participates in the destructive green tide observed all over the world. However, *U. lactuca* has been found to contain commercially valuable constituents including bioactive compounds, food or biofuel (Dominguez and Loret, 2019). Zhu et al. (2017) prepared selenium nanoparticles decorated with *U. lactuca* polysaccharide ULP with average size ∼130 nm. ULP treatment exhibited a prominent protective effect on DSS-induced acute colitis in mice. ULP could suppress macrophage infiltration by decreasing the colonic CD68 level. Moreover, ULP markedly attenuated oxidative stress damage *via* increasing the colonic GSH, GPx, and Se levels and reducing the generation of MDA. In addition, ULP exerted a strong effect on mitigating the mRNA expression and levels of inflammatory markers, including IL-6, TNF-α, iNOS, and COX-2 in DSS-induced colitis mice and in LPS-stimulated BMDMs. Mechanistically, ULP pretreatment was found to suppress the inactivation of the NF-κB signaling pathway *via* retarding the phosphorylation of NF-κB p65 and IκBα *in vivo* and *in vitro*. Altogether, these data indicated that ULP supplementation might be a potential therapeutic candidate for preventing inflammatory diseases, like IBD.

### Eucheuma cottonii

The edible red algae *E. cottonii* is largely cultivated due to carrageenan production. Meanwhile, it is rich in active constituents, such as polyphenols, steroids, and polysaccharides (Namvar et al., 2012). Sudirman et al. (2018) extracted crude polysaccharides ECP from *E. cottonii* and found that after treatment for 7 days, ECP could remarkably ameliorate DSS-induced colitis by alleviating weight loss and colonic mucosa injury and reducing the ratio of colon weight and length. Additionally, ECP also dramatically decreased the levels of pro-inflammatory mediators TNF-α, IL-1β, and IL-6 in serum and increased the anti-inflammatory cytokine IL-10 level in colon tissue of DSS-treated mice. This result revealed that ECP exhibited a pronouncedly effect on colitis, which might be used to a promising candidate for the treatment of IBD in the future.

### Blidingia minima

*B. minima* is prevalent along many coastlines since these green algae originate in areas with large differences in seawater salinity and freshwater tidal areas. Song et al. (2019) extracted polysaccharides from *B. minima*, and conducted the *in vitro* and *in vivo* experiments to investigate the anti-inflammatory effect of BMP through using DSS-treated intestinal epithelial cells (IPEC-J2 cells) and C57BL/6J mice, respectively. Results illustrated that BMP exhibited a remarkably anti-inflammatory effect on DSS-stimulated cells. Additionally, BMP noticeably ameliorated colitis symptoms, and improved colonic infiltration by reducing the levels of MPO and EPO in the colonic tissue. BMP treatment restored the colonic barrier functions by upregulating the expression of TJs proteins (ZO-1, occludin, and claudin-1) and DAO activity, and downregulating the ET-1 level in DSS-treated mice. Moreover, the mRNA expression and production of pro-inflammatory cytokines TNF-α and IL-1β were mitigated, while anti-inflammatory factor IL-10 was enhanced after BMP treatment. Furthermore, the phosphorylated protein levels of NF-κB, IκBα, and AKT in colonic tissue were all decreased by BMP supplementation. These results indicated that BMP was endowed with a pronounced anti-inflammatory property, and its mechanism might be intimately associated with the inhibition of NF-κB and AKT pathways.

## Discussion and Conclusion

**FIGURE 4:**
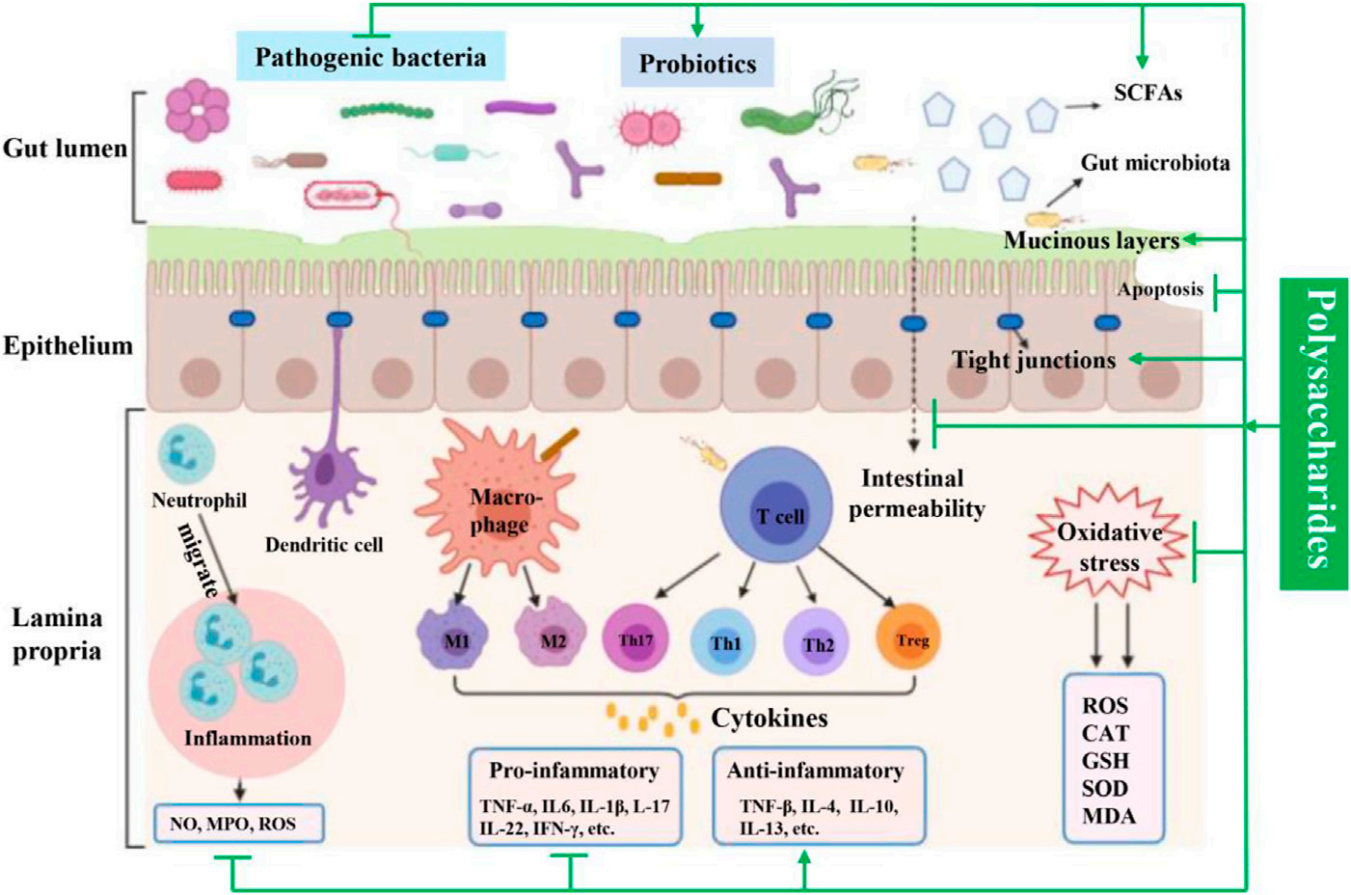
Involved mechanisms of natural-derived polysaccharides in the treatment of IBD (promotion and inhibition).

With the progress of civilization, people's lifestyles and eating habits have also undergone tremendous changes. The diet structure is changing to a high-fat, low-fiber pattern, which has a negative impact on the body, decreases immunity, and makes people susceptible to diseases and pathogenic bacteria. Early IBD was prevalent in western countries. However with globalization and changes in lifestyle, the incidence of IBD is rapidly increasing in Asian countries such as India and China (Mak et al., 2020). Current strategies for IBD therapy are mainly synthetic drugs intervention, which is accompanied by a wide range of side effects. For centuries, polysaccharides, as a natural product, are endowed with various beneficial treatment effects, such as immunomodulatory, anti-inflammatory, antioxidant, and bacteriostasis, with no or fewer toxicity, making them one of the novel therapeutic agents or optimal candidate drugs for treating IBD. In this review article, for the first time, we present the investigations conducted in this field since the beginning of this century, mainly focusing on polysaccharides from plants, edible mushrooms, and seaweeds that exert ameliorative effects against experimental IBD and related properties along with their mechanisms of action (Figure 4).

Intestinal epithelium, located at the junction of intestinal microbiota and lymphoid tissue, plays a key role in mucosal formation and defense against luminal antigens, endotoxins, and pro-inflammatory mediators. The barrier defects are considered as the first event during IBD pathological progression, resulting in increasing intestinal permeability, and ultimately leading to a severe inflammatory response in intestinal tissues (Luo et al., 2020). Mucinous layer, consists of mucins (mainly including MUC1 and MUC2) released by epithelial goblet cells, is the first line of defense against the invasion of pathogens. TJs proteins (including ZO-1, ZO-2, occludin, and claudin1), located between the adjacent intestinal epithelial cells, are an essential mechanical barrier, which plays a crucial role in the regulation of intestinal epithelial barrier integrity and intestinal permeability (Li C. L. et al., 2020; Zhao et al., 2020). In IBD patients, decreased expression of TJs and mucins were observed, which are currently emerged as a novelty effective target for IBD therapy. In this review, we found that the expression of TJs proteins and mucins proteins was reduced by DSS or TNBS exposure. However, this change was reversed after polysaccharides treatment, such as ASP, WJPs, LEP, and BMP.

In addition to TJs changes, increased epithelial cell apoptosis may also lead to intestinal barrier dysfunction (Dong et al., 2015). Under physiological conditions, the life span of intestinal epithelial cells is relatively short. Apoptosis caused by programmed cell death is a carefully controlled process, which is essential for maintaining normal barrier function (Yuan et al., 2015; Butkevych et al., 2020). However, excessive epithelial cell apoptosis can damage the intestinal barrier function and is related to the initiation and development of IBD (Wang W. et al., 2020). The Bcl-2 family plays a vital role in the apoptosis pathway, and Bcl-2 is an apoptosis suppressor molecule involved in protection against oxidative stress-induced apoptosis and maintenance of cell survival. Conversely, Bax is an apoptosis-promoting gene. The ratio of Bax/Bcl-2 determines cell growth or apoptosis (Jia et al., 2015). Additionally, Caspase-3 is an important downstream transducer in most types of cells. It plays an important role in the initiation of apoptosis by cleavage of cell substrates (Gansukh et al., 2019). In the current review, we found that the expression of Bax and caspase-3 was significantly downregulated, whereas the expression of Bcl-2 was upregulated by multiple polysaccharides treatment, such as ASP, MAP, EP-1, and DIP, leading to a decrease of Bax/Bcl-2 ratio. These results elucidated that these polysaccharides exerted a certain anti-apoptotic effect in DSS- or acetic acid–induced UC mice, which promote the recovery of intestinal barrier defects.

Abnormal chronic inflammation is one of the pivotal clinical symptoms of IBD (Na et al., 2019). In the presence of persistent inflammation, strong colonic inflammation was developed, accompanied with augmented levels of pro-inflammatory mediators (e.g., TNF-α, IL-1β, and IL-6) and descended production of anti-inflammatory cytokines (e.g., IL-4, IL-10, and IL-13), causing increase of intestinal permeability and impairment of mucosal barrier function. Among them, pro-inflammatory mediators could promote the immune inflammatory response through activating Th cells, triggering neutrophil migration and inducing intracellular signal transduction. Inversely, anti-inflammatory cytokines could ameliorate the development of colitis clinically (Lee et al., 2020). Therefore, regulating the balance of pro-inflammatory and anti-inflammatory cytokines is a crucial therapeutic strategy to suppress the inflammatory process. In the current review, the up-regulated release of pro-inflammatory mediators, such as TNF-α, IL-6, IFN-γ, IL-1β, and IL-18, and the down-regulated levels of anti-inflammatory factors, such as IL-4, IL-10, and IL-13, in colitis mice were dramatically inhibited by polysaccharides from edible and medicinal plants (e.g., APS, DOPS, and ASPP), edible mushrooms (e.g., HECP, EP-1, and IOP), and seaweeds (e.g., ECP, BMP, and GBP). MPO activity, a marker of the inflammatory response in damaged tissues, was also decreased. Further mechanism study manifested that their anti-inflammatory effect was mainly associated with the blockage of major inflammatory signaling pathways including NF-κB, MAPK, IL-6/JAK2/STAT3, PI3K/AKT, and NLRP3 inflammasome pathways.

In addition, oxidative stress in intestinal mucosa has been regarded as a key event during the pathological progression of IBD (Piechota-Polanczyk and Fichna, 2014). Excessive inflammatory cytokines could induce oxidative stress and nitrosative stress by irritating ROS and RNS production systems, which in turn further aggravates the immune inflammation, resulting in tissue damage and deterioration of patient’s condition (Saber et al., 2019). In the development of IBD, ROS-generating processes are sharply elevated, consequently causing intestinal epithelial cell injury (Mei et al., 2020). Therefore, the anti-oxidative stress approach is one of the crucial strategies in treating IBD. In the experimental IBD model, excessive oxidative stress was observed as the increased contents of ROS, MDA, NO_3_/NO_2_, decreased levels of SOD, CAT, and T-AOC. However, after the treatment of polysaccharide, such as PLS, HECP, EP-1, and LEP, these changes could significantly be reversed.

Gut microbiota, approximately 100 trillion microorganisms, is a large bacterial community that colonizes in the intestine, which plays a substantial role in regulating the intestinal structure and intestinal permeability, and its dysbiosis is closely related to the IBD (Stange and Schroeder, 2019). In the case of IBD, the richness and diversity of intestinal microbiota were strikingly reduced in comparison with healthy individuals. Moreover, the abundance of pathogenic bacteria, such as *Escherichia*, *Enterococcus,* and *Prevotella* was increased while the ratio of *Firmicutes* to *Bacteroidetes* decreased dramatically (Liu W. et al., 2018). In the present review, we found that natural polysaccharides, including CP, ALP-1, ASPP, and HECP prominently increased the diversity of intestinal flora and modulated the gut microbiota community close to that of normal mice.

In addition, SCFAs, mainly including AA, PA, BA, and VA, are the major fermentation products of prebiotic metabolism in the gut lumen, which plays a crucial function in intestinal homeostasis (Sun et al., 2017). Notably, AA and BA can activate GPR41 and GPR43 and suppress histone deacetylase to exhibit the anti-inflammatory effect (Kobayashi et al., 2017). In colonic tissues of IBD patients, the contents of BA, AA and so on, were significantly decreased (Macia et al., 2015). Thus, the regulation of SCFAs production has also become a promising therapeutic target for IBD treatment. Interesting, in the present review, it showed that the decreased proportions of AA, BA, PA, and TA in colon of the disease model group were remarkably upregulated by polysaccharides treatment, including ASPP, GLP, EP-1, and FVP.

In summary, this article firstly summarizes the therapeutics effect of polysaccharides derived from plants, mushrooms, and seaweeds and shows how these polysaccharides exhibited prominently and beneficial effects in attenuate colonic inflammation. Multiple factors are involved in their protective effects on IBD, including the alleviation of the intestinal barrier destruction *via* increasing TJs proteins and mucus layer protective proteins and reducing epithelial cell apoptosis, modulation of gut microbiota, reduction of excessive oxidative stress, and inhibition of aberrant inflammatory response, inflammatory cells infiltration, and MPO activity through blocking NF-κB, MAPK, IL-6/JAK2/STAT3, PI3K/AKT, and NLRP3 inflammasome signaling pathways. In conclusion, these findings provided a solid scientific basis for utilization of natural polysaccharides from plants, mushrooms, and seaweeds as promising candidates for IBD therapy. Further clinical and preclinical studies to reveal the molecular mechanisms are needed to promote the clinical translation of natural polysaccharides for IBD.

## Glossary

AA: Acetic acid
Akt: Protein kinase B
AMPK: AMP-activated protein kinase
Ara: Arabinose
ASC: Apoptosis-associated speck-like protein containing a CARD
ATF6: Activating transcription factor 6
ATP: Adenosine triphosphate
BA: Butyrate acid
Bax: Bcl2-associated X protein
Bcl-2: B-cell lymphoma-2
BDP: Beclomethasone dipropionate
Bip: Binding immunoglobulin protein
BMDMs: Bone marrow-derived macrophages
B3gnt6: Beta-1,3-N-Acetylglucosaminyltransferase 6
B4galnt2: Beta-1,4-N- acetylgalactosaminyltransferase 2
CAT: Catalase
CA1: Carbonic anhydrase I
C-Cas3: Cleaved-caspase-3
Ccl3: C-C motif chemokine 3
Ccl5: C-C motif chemokine 5
Cd3e: T-cell surface glycoprotein CD3 epsilon chain
Cd8a: T-cell surface glycoprotein CD8 alpha chain
CDH1: Cadherin 1
CFC-113: Trichlorotrifluoroethane
CHOP: C/EBP homologous protein
COX-2: Cyclooxygenase-2
C3: Complement C3
DAI: Disease activity index
DAO: Diamine oxidase
DHT: Dihydrotestosterone
DSS: Dextran sulfate sodium
EPO: Eosinophil peroxidase
ER: Endoplasmic reticulum
ERK: Extracellular signal-regulated kinase
ET-1: Endothelin 1
EtOH: Ethanol
Fabp2: Fatty acid binding protein 2
FD-4: FITC-conjugated dextran-4
Foxp3: Transcription factor forkhead box P3
FP: Fractional precipitation
Fru: Fructose
F4/80: Adhesion G protein-coupled receptor E1
Gal: Galactose
GalA: Galacturonic acid
GATA3: GATA-binding protein 3
Glc: Glucose
GluA: Glucuronic acid
GPC: Gel permeation chromatography
GPR41: G protein-coupled receptor 41
GPR43: G protein-coupled receptor 43
GPx: Glutathione peroxidase
Gro: Cxcl1/2/3
GSH: Glutathione
Hmgcs2: 3-hydroxy-3-methylglutaryl-CoA synthase 2
HNF4-α: Hepatocyte nuclear factor 4-α
HO-1: Heme oxygenase
Hp: Haptoglobin
IBA: Isobutyric acid
IBD: Inflammatory bowel disease
ICAM-1: Intercellular adhesion molecule-1
IEC: Ion-exchange chromatography
IFN-γ: Interferon γ
IgA: Immunoglobulin A
IgM: Immunoglobin M
IκBα: Inhibitor of nuclear factor-κB
IKK: Inhibitor of nuclear factor-κB kinase
IL: Interleukin
IL-22BP: IL-22binding protein
IL-12p40: Interleukin 12B
Il21r: Interleukin 21 receptor
iNOS: Inducible nitric oxide synthase
IRE1α: Inositol-requiring enzyme 1α
IRF4: Interferon regulatory factor 4
IRF5: Interferon regulatory factor 5
IVA: Isovaleric acid
JAK2: Janus kinase 2
JNK: C-Jun N-terminal kinase
Lck: Lymphocyte cell-specific protein tyrosine kinase
LC3: Light chain 3
Ly6G: Lymphocyte antigen 6 complex locus protein G
LPS: Lipopolysaccharide
Man: Mannose
MAPK: Mitogen-activated protein kinases
MCP-1β: Monocyte chemoattractant protein-1β
MDA: Malondialdehyde
Mhc2: MHC class II antigen
MMP: Mitochondrial membrane potential
MPO: Myeloperoxidase
MUC1: Mucin 1
MUC2: Mucin 2
MW: Molecular weight
NA: Not Available
NF-κB: Nuclear factor -κB
NLRP3: (NOD)-like receptor family pyrin domain containing 3
NO: Nitric oxide
NOx: Nitrites
NO3/NO2: Nitrate/nitrite
NSAIDs: Nonsteroidal anti-inflammatory drugs
OCR: Oxygen consumption rate
OFR: Oxygen free radical
PA: Propionic acid
p-ACC: Phospho-acetyl-coA carboxylase
PCNA: Proliferating cell nuclear antigen
PERK: Protein kinase RNA-like ER kinase
p-ERK: Phospho-extracellular signal-regulated kinase
PGE2: Prostaglandin E2
p-IκBα: Phosphonated inhibitor of nuclear factor-κB
PI3K: Phosphatidylinositol-3-kinase
p-JNK: Phospho-c-jun N-terminal kinase
p62: Sequestosome 1
p65: Nuclear factor-κB p65
p-p38: Phospho-p38 mitogen-activated protein kinases
p-p65: Phospho-nuclear factor-κB-p65
p-STAT3: Phospho-signal transducer and activator of transcription 3
Ptgs2: Prostaglandin-endoperoxide synthase 2
Reg3β: Regenerating islet-derived 3β
Reg3γ: Regenerating islet-derived 3γ
Relmβ: Resistin-like molecule β
Rha: Rhamnose
Rib: Ribose
RNS: Reactive nitrogen species
ROR-γt: Retinoid-related orphan nuclear receptor γt
ROS: Reactive oxygen species
SCFAs: Short chain fatty acids
Se: Selenium
SOD: Superoxide dismutase
STAT3: Signal transducer and activator of transcription 3
T-AOC: Total antioxidant capacity
TCA: Trichloroacetic acid
T-bet: T-box protein 21
TER: Transepithelial electrical resistance
TFF3: Trefoil factor 3
Th: Helper T cells
TJs: Tight junctions
TJP1: Tight junction protein 1
TLR4: Toll-like receptor 4
TNBS: Trinitrobenzene sulfonic acid
TNF-α: Tumor necrosis factor α
Trbv: T-cell receptor beta chain V region;
Treg: Regulatory T cell
TUNEL: Terminal deoxynucleotidyl transferase-mediated dUTP nick end labeling
UA: Uronic acid
ULK1: Unc51-like kinase 1
VA: Valeric acid
XBP1: X-box binding protein 1
Xyl: Xylose
ZO-1: Zonula occluden-1
